# Palmitoylation: A Fatty Regulator of Myocardial Electrophysiology

**DOI:** 10.3389/fphys.2020.00108

**Published:** 2020-02-19

**Authors:** Kobina Essandoh, Julie M. Philippe, Paul M. Jenkins, Matthew J. Brody

**Affiliations:** ^1^Department of Pharmacology, University of Michigan, Ann Arbor, MI, United States; ^2^Department of Psychiatry, University of Michigan, Ann Arbor, MI, United States; ^3^Department of Internal Medicine, University of Michigan, Ann Arbor, MI, United States

**Keywords:** S-acylation, palmitoylation, zDHHC enzymes, cardiac physiology, electrophysiology, post-translational modifications, ion channels, myocardium

## Abstract

Regulation of cardiac physiology is well known to occur through the action of kinases that reversibly phosphorylate ion channels, calcium handling machinery, and signaling effectors. However, it is becoming increasingly apparent that palmitoylation or S-acylation, the post-translational modification of cysteines with saturated fatty acids, plays instrumental roles in regulating the localization, activity, stability, sorting, and function of numerous proteins, including proteins known to have essential functions in cardiomyocytes. However, the impact of this modification on cardiac physiology requires further investigation. S-acylation is catalyzed by the zDHHC family of S-acyl transferases that localize to intracellular organelle membranes or the sarcolemma. Recent work has begun to uncover functions of S-acylation in the heart, particularly in the regulation of cardiac electrophysiology, including modification of the sodium-calcium exchanger, phospholemman and the cardiac sodium pump, as well as the voltage-gated sodium channel. Elucidating the regulatory functions of zDHHC enzymes in cardiomyocytes and determination of how S-acylation is altered in the diseased heart will shed light on how these modifications participate in cardiac pathogenesis and potentially identify novel targets for the treatment of cardiovascular disease. Indeed, proteins with critical signaling roles in the heart are also S-acylated, including receptors and G-proteins, yet the dynamics and functions of these modifications in myocardial physiology have not been interrogated. Here, we will review what is known about zDHHC enzymes and substrate S-acylation in myocardial physiology and highlight future areas of investigation that will uncover novel functions of S-acylation in cardiac homeostasis and pathophysiology.

## Introduction

S-acylation or palmitoylation is the post-translational modification of protein cysteines with saturated fatty acids that imparts spatiotemporal regulation of protein hydrophobicity and thereby can modulate many aspects of protein function including localization, activity, interactions with cofactors, membrane topology, and stability (Linder and Deschenes, 2007; Greaves and Chamberlain, 2011; Chamberlain and Shipston, 2015). S-acylation is unique amongst lipid modifications on proteins in that it is a highly regulated and reversible process controlled by 23 Asp-His-His-Cys (DHHC) S-acyltransferases (Fukata et al., 2004; Greaves and Chamberlain, 2011; Gottlieb and Linder, 2017). Certain serine hydrolases, including acyl protein thioesterase-1 and -2 and α/β-Hydrolase domain-containing proteins (ABHD10, 17A, 17B, and 17C) exhibit thioesterase activity and function as protein S-deacylases (Hirano et al., 2009; Tomatis et al., 2010; Lin and Conibear, 2015a, b; Yokoi et al., 2016; Cao et al., 2019). This tight enzymatic control enables S-acylation to be acutely altered in response to stimuli or changes in cellular environment and rapidly regulate cellular and physiological responses.

**FIGURE 1:**
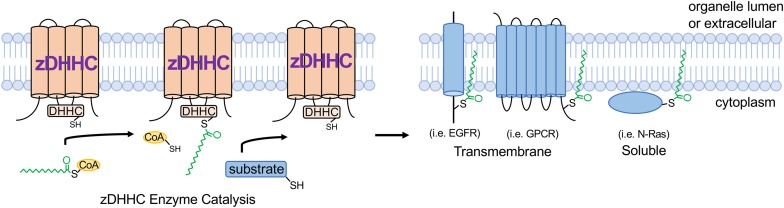
zDHHC S-acyltransferase-mediated substrate modification. zDHHC enzymes modify substrates through a “ping-pong” kinetic mechanism (Jennings and Linder, 2012) whereby the enzyme transiently accepts an acyl chain on its DHHC Cys from acyl-CoA and then transfers the acyl group onto the substrate Cys residue. zDHHCs are transmembrane proteins with their enzymatic DHHC motif on the cytoplasmic loop and substrates are S-acylated on cytoplasmic juxtamembrane or membrane-associated Cys residues. *GPCR*, G-protein-coupled receptor; *EGFR*, epidermal growth factor receptor; *CoA*, coenzyme A.

The term palmitoylation is frequently used interchangeably with S-acylation and while the 16-carbon fatty acid palmitate (16:0) is most commonly attached to protein cysteines, some zDHHC enzymes are more promiscuous and will transfer shorter (14:0) or longer (18:0) length acyl chains onto substrates (Chamberlain and Shipston, 2015; Greaves et al., 2017; Rana et al., 2018). zDHHC S-acyltransferases are polytopic transmembrane proteins, many of which localize to the Golgi or endoplasmic reticulum (ER) but with some also localizing to the plasma membrane, endomembrane system, or intracellular vesicles (Ohno et al., 2006; Korycka et al., 2012). Although some zDHHCs have overlapping substrates, they exhibit a high degree of substrate specificity that is mediated in part by protein sequence recognition elements on the substrate and zDHHC enzymes, tissue- and temporal-specific expression patterns, and subcellular localization (Linder and Deschenes, 2007; Tsutsumi et al., 2008; Howie et al., 2014; Lemonidis et al., 2014; Plain et al., 2017; Shah et al., 2019). Catalysis of S-acylation by zDHHC enzymes occurs via a “ping-pong” kinetic mechanism in which the zDHHC enzyme undergoes transient autoacylation on its DHHC domain cysteine residue and subsequently transfers the fatty acid onto the cysteine of the protein substrate (Jennings and Linder, 2012; Rana et al., 2018; Figure 1). Importantly, many zDHHC enzymes are abundantly expressed in heart (Howie et al., 2014), suggesting vital regulatory functions in myocardial physiology. S-deacylating thioesterases are predominantly cytoplasmic enzymes and are also expressed in the heart, but far less is known regarding their substrate specificity and physiological functions (Davda and Martin, 2014; Chamberlain and Shipston, 2015).

While some substrates exhibit relatively stable S-acylation to mediate trafficking to and/or association with specific membrane domains, other proteins, including signaling molecules such as G-proteins and small GTPases, undergo rapid cycles of S-acylation and deacylation that can control movement to specific intracellular membrane microdomains with remarkable temporal resolution (Martin and Cravatt, 2009; Tsutsumi et al., 2009; Martin et al., 2012; Won and Martin, 2018). Indeed, the functional effects and kinetics of protein S-acylation are substrate and context specific. For example, the tyrosine kinase c-Met is S-acylated in the ER shortly after biosynthesis as a stable modification to promote anterograde trafficking to the plasma membrane (Coleman et al., 2016) while S-acylation of the ER-resident chaperone calnexin occurs several hours after synthesis to enhance protein stability and thereby deploy nascent calnexin protein at the ER membrane (Lakkaraju et al., 2012; Dallavilla et al., 2016). In contrast, H-Ras and N-Ras undergo rapid minute timescale cycles of deacylation/reacylation to regulate shuttling between the Golgi apparatus and signaling domains at the plasma membrane (Rocks et al., 2005) underscoring the need for further investigation of protein S-acylation dynamics and functional consequences, especially in physiologically relevant cell types such as cardiomyocytes. Recent studies demonstrate a critical role for S-acylation in many pathologies including several cancers (Liu et al., 2016; Runkle et al., 2016; Chen et al., 2017; Kharbanda et al., 2017; Chen S. et al., 2019), inflammatory diseases (Beard et al., 2016; Mukai et al., 2016; Hansen et al., 2019), and neurological disorders (Mukai et al., 2004; Cho and Park, 2016; Sanders et al., 2016; Kouskou et al., 2018). Moreover, mutations in *ZDHHC9* are associated with X-linked intellectual disability (Raymond et al., 2007; Han et al., 2017), suggesting a direct link between defective S-acylation and human disease. Here we will focus on the functions of S-acylation, zDHHC enzymes, and modified substrates in the heart, but comprehensive reviews can be found elsewhere (Chamberlain and Shipston, 2015; Jiang et al., 2018).

Cardiomyocytes, which comprise 70–90% of the volume fraction of the heart (Reiss et al., 1996; Zhou and Pu, 2016), are very specialized, electrically excitable contractile cells that mediate the predominant cardiac function of pumping blood to the peripheral tissues and organs. Importantly, the cardiomyocyte cytoplasm is packed full with myofilaments and mitochondria, which occupy approximately 60% and 30% of the intracellular milieu, respectively (Barth et al., 1992; Piquereau et al., 2013), leaving limited free cytoplasmic space for signaling molecules and membrane proteins to navigate and traffic. It is within this complex cytoarchitecture that membrane proteins, including ion channels and receptors, must traffic to the appropriate membrane microdomains, and signaling molecules must navigate to assemble into signaling complexes that nucleate at specific intracellular membranes. Beyond providing lipid-based molecular instructions to direct proteins to specific membranes, S-acylation can also locally alter how strongly a protein interacts with membranes or the topology of a membrane protein within a given cellular membrane, which dramatically affects protein activity as has been demonstrated for many ion channels (Chaube et al., 2014; Reilly et al., 2015; Pei et al., 2016; Duncan et al., 2019), receptors (Runkle et al., 2016; Chen et al., 2017; Kharbanda et al., 2017), and kinases (Barylko et al., 2009; Zhou et al., 2014; Akimzhanov and Boehning, 2015; Figure 1).

zDHHC mouse models suggest important roles for these enzymes in the heart. Deletion of the ER- and Golgi-localized enzyme zDHHC16 results in defects in eye development and perinatal cardiomyopathy and lethality (Zhou et al., 2015; Abrami et al., 2017). In contrast, cardiac muscle lacking the plasma membrane enzyme zDHHC5 exhibits enhanced recovery of contractile function following anoxia (Lin et al., 2013). Moreover, mutation of an S-acylation site (C981) in the cardiac voltage-gated sodium channel (Na_v_1.5) is associated with cardiac arrhythmia in human patients (Kapplinger et al., 2009; Pei et al., 2016). Although genetic deletion of both acyl protein thioesterase-1 and -2 in mice did not result in an overt phenotype, cardiac function was not evaluated (Won and Martin, 2018). Pharmacological or genetic strategies to inhibit or augment specific S-acylation events in cardiomyocytes could provide novel therapeutic interventions for the treatment of heart disease.

S-acylation undoubtedly plays fundamental roles in cardiac function and disease, including modulation of ion channel function and signal transduction in cardiac myocytes. Here, we will review how S-acylation modulates myocardial physiology with a focus on the substrates and modifications demonstrated to impact cardiomyocyte electrophysiology as well as highlight other areas of cardiac physiology regulated by S-acylation that warrant future investigation.

## Myocardial Electrophysiology

**FIGURE 2:**
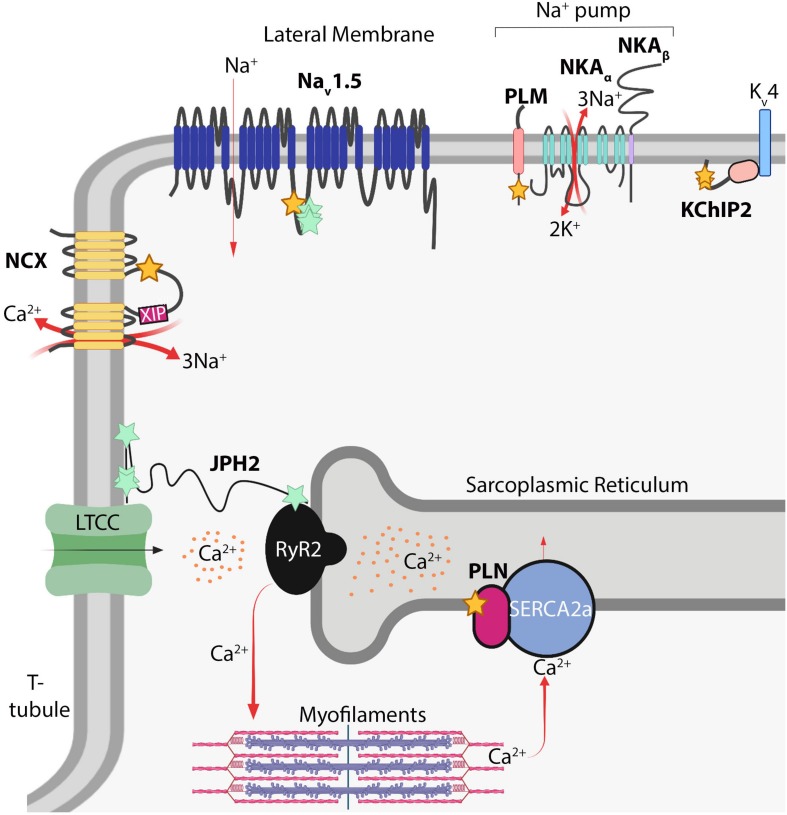
S-acylation in myocardial electrophysiology. Schematic of S-acylated ion channels, transporters, and auxiliary proteins in cardiomyocytes. Yellow stars denote cysteines modified by S-acylation; turquoise stars denote potential modification sites. *NCX*, Na^+^/Ca^2+^-exchanger; *XIP*, exchanger inhibitory peptide; *Na_v_1.5*, voltage-gated Na^+^ channel; *PLM*, phospholemman; *NKA*, Na^+^/K^+^-ATPase; *KChIP2*, K^+^ channel interacting protein 2; *K_v_4*, delayed rectifier voltage-gated K^+^ channel; *PLN*, phospholamban; *RyR2*, ryanodine receptor 2; *SERCA2A*, sarcoendoplasmic reticulum ATPase 2A; *JPH2*, junctophilin-2; *LTCC*, voltage-gated L-type Ca^2+^ channel. Image created with BioRender (www.biorender.com).

Despite the lack of knowledge of the functions of S-acylation in cardiomyocytes relative to other cell types such as neurons (Fukata and Fukata, 2010; Matt et al., 2019), recent studies demonstrate cardiomyocyte electrophysiology is highly regulated by this modification. Cardiomyocyte function is principally regulated by local ion concentrations that establish membrane potential and stimulate myofilament contraction. Rapid influx of Na^+^ ions depolarizes the cardiomyocyte and is followed by Ca^2+^ influx through voltage-dependent L-type calcium channels (Ca_v_1.2) that stimulate ryanodine receptor 2 (RyR2) to release Ca^2+^ from internal stores in the sarcoplasmic reticulum (SR). This, in turn, raises cytosolic Ca^2+^ levels by an order of magnitude to directly activate myofilament contraction in a process termed excitation-contraction coupling (MacLennan and Kranias, 2003; Fearnley et al., 2011; Ljubojevic and Bers, 2015). Cytosolic Ca^2+^ is returned to basal diastolic levels by reuptake back into the SR through the sarco ER ATPase (SERCA2a), and to a lesser extent, extrusion of Ca^2+^ outside the cell by the Na^+^/Ca^2+^-exchanger (NCX) (MacLennan and Kranias, 2003; Lytton, 2007; Figure 2). Cardiomyocyte electrophysiological properties are highly regulated by a number of ion channels, pumps, and auxiliary proteins each optimally positioned within the cardiomyocyte to impart precise spatiotemporal control of intracellular Na^+^, Ca^2+^, and K^+^ ion concentrations (MacLennan and Kranias, 2003; Lytton, 2007; Fuller et al., 2013; Brody and Lee, 2016; Edokobi and Isom, 2018; Woon et al., 2018). Here we will highlight S-acylation-dependent mechanisms controlling ion channel homeostasis and excitation-contraction coupling in cardiomyocytes.

## Voltage-Gated Sodium Channel (Na*_v_*1.5)

Inward Na^+^ current provides the membrane depolarization responsible for action potential generation in cardiomyocytes. This occurs through voltage-gated sodium channels composed of the pore-forming Na_v_1.5 α-subunit, which contains four homologous domains of six transmembrane domains each, and two auxiliary β subunits containing a single transmembrane domain and extracellular immunoglobulin loop (Detta et al., 2015; Edokobi and Isom, 2018). Na_v_1.5 (encoded by *SCN5A*) is the major regulator of cardiac excitation and mutations in Na_v_1.5 are associated with a number inherited arrhythmogenic channelopathies and disorders including Brugada syndrome, long QT syndrome, and atrial fibrillation (Abriel, 2007; Detta et al., 2015). A C981F mutation in Na_v_1.5 was reported in a patient with long QT syndrome (Kapplinger et al., 2009; Pei et al., 2016), which corresponds to an intracellular loop cysteine on the domain II-III linker region (Figure 2), a location ideally situated to regulate pore activity. Na_v_1.5 is S-acylated in the heart and mutation of four cysteines (including Cys981) in this region abrogated S-acylation (Pei et al., 2016), suggesting Na_v_1.5 may by lipidated at multiple sites on this intracellular loop. More importantly, the C981F mutation or pharmacological inhibition of Na_v_1.5 S-acylation did not affect cell surface expression but instead augmented closed-state inactivation and reduced channel availability and cardiomyocyte excitability (Pei et al., 2016). In contrast, increasing S-acylation of Na_v_1.5 prolonged action potential duration and induced early after depolarizations (Pei et al., 2016), revealing S-acylation functions as a rheostat for Na_v_1.5 channel gating and cardiac excitability.

## Na^+^ Pump (Na^+^/K^+^-ATPase)

The Na^+^ pump establishes the resting membrane potential in cardiomyocytes through energy-dependent extrusion of three Na^+^ ions concomitant with import of two K^+^ ions. This homeostatic maintenance of intracellular Na^+^ and K^+^ concentrations sets the electrochemical gradient for Na_v_1.5, other Na^+^-dependent transporters, and NCX (Howie et al., 2013; Pavlovic et al., 2013). The cardiac Na^+^ pump consists of the Na^+^/K^+^-ATPase catalytic α subunit (α1 or α2), the β1 subunit, and an ancillary FXYD protein called phospholemman (Fuller et al., 2013; Figure 2). The α and β subunits of the Na^+^ pump are S-acylated in non-cardiac cells (Kang et al., 2008; Forrester et al., 2011; Martin et al., 2012; Howie et al., 2013), yet targeted studies investigating the functional consequences of these modifications or whether they occur in the heart have not been reported. However, recent studies have uncovered a vital role for S-acylation of phospholemman in regulating cardiac Na^+^ pump activity. Phospholemman, a small 72 amino acid transmembrane protein that negatively regulates Na^+^ pump activity, is palmitoylated on its intracellular membrane proximal residues, Cys40 and Cys42, adjacent to the Na^+^/K^+^-ATPase α subunit (Tulloch et al., 2011). In cardiomyocytes, S-acylation of phospholemman occurs predominantly at Cys40 (Figure 2) by the action of the sarcolemmal enzyme zDHHC5, which is required for phospholemman-mediated repression of Na^+^ pump activity (Howie et al., 2014). Thus, regulation of phospholemman by zDHHC5 provides an S-acylation-dependent mechanism to acutely fine tune the cardiac Na^+^ pump.

## Na^+^/Ca^2+^-Exchanger (NCX)

The NCX is a sarcolemmal ion transporter containing 10 transmembrane domains that extrudes Ca^2+^ into the extracellular space in exchange for import of three Na^+^ ions, which along with reuptake of Ca^2+^ into the SR by SERCA2a, restores cytosolic Ca^2+^ levels following myofilament contraction (Figure 2). NCX activity thus is an important homeostatic regulator of excitation-contraction coupling. Reduced NCX activity and consequent diminished removal of systolic Ca^2+^ can elevate SR Ca^2+^ stores and be a source of arrhythmogenesis (Lytton, 2007; Voigt et al., 2012; Fuller et al., 2016). In contrast, enhanced NCX expression, which occurs in heart failure, can hasten diminished contractile function by reducing SR Ca^2+^ stores available for excitation-contraction coupling and contribute to arrhythmogenic delayed after depolarizations triggered by increased NCX-mediated Na^+^ influx concomitant with Ca^2+^ efflux (Lytton, 2007; Ottolia et al., 2013). NCX can also function in reverse-mode depending on Na^+^ and Ca^2+^ concentration gradients, thereby extruding Na^+^ and importing Ca^2+^, which can exacerbate Ca^2+^ overload-induced cardiomyocyte necrosis during ischemia (Chen and Li, 2012; Fuller et al., 2016). Therefore, tight regulation of NCX activity is required for proper cardiomyocyte contraction, electrophysiology, and prevention of cardiac arrhythmias. NCX1, the only isoform expressed in cardiomyocytes, is S-acylated in the heart, brain and kidney (Reilly et al., 2015). NCX1 is S-acylated at the Golgi exclusively on Cys739 on the intracellular regulatory f-loop between the fifth and sixth transmembrane domains (Figure 2), tethering the cytoplasmic f-loop to the membrane (Reilly et al., 2015; Fuller et al., 2016). While S-acylation does not modulate localization or trafficking of NCX1 to the sarcolemma, it is required for effective inactivation in response to reduced intracellular Ca^2+^ or phosphatidylinositol 4,5-bisphosphate (PIP2) levels (Reilly et al., 2015; Fuller et al., 2016), physiological cues that trigger NCX1 inactivation (Lytton, 2007). Inactivation of NCX1 requires the action of an autoinhibitory peptide, the exchanger inhibitory peptide (XIP), located at the juxtamembrane N-terminal side of the cytosolic regulatory f-loop (amino acids 219-238) of NCX1 (Figure 2). S-acylation of NCX1 at Cys739 on the C-terminal side of this f-loop appears to modify its structure to enhance XIP-mediated inactivation of NCX1 (Molinaro et al., 2015; Reilly et al., 2015; Fuller et al., 2016; Figure 2). Therefore, S-acylation of NCX1 at the cardiomyocyte Golgi modulates its activity at the sarcolemma by controlling its propensity for inactivation in response to physiological stimuli (Reilly et al., 2015; Fuller et al., 2016). Since roughly 60% of NCX1 is S-acylated in the heart (Reilly et al., 2015; Fuller et al., 2016), the stoichiometry and dynamics of NCX1 S-acylation are likely to have important consequences for cardiac contractile function and arrhythmogenesis.

## KChIP2

In addition to Na^+^ and Ca^2+^ currents, K^+^ currents mediated by voltage-gated K^+^ channels (K_v_ channels) are precisely regulated and make up an instrumental regulatory component of cardiac electrophysiology. K^+^ channel interacting protein 2 (KChIP2) primarily functions as an accessory K_v_4 channel protein and is required for trafficking and cell surface expression of K_v_4.2 and Kv4.3 and the generation of the transient outward K^+^ current in cardiomyocytes (Kuo et al., 2001; Takimoto et al., 2002; Shibata et al., 2003; Grubb et al., 2012). KChIP2 is a cytoplasmic protein with four EF hand motifs that bind Ca^2+^ and S-acylation sites at Cys45 and Cys46 that are required for its localization to the cell membrane in HEK cells (Takimoto et al., 2002; Grubb et al., 2012; Figure 2). Importantly, Cys45 and Cys46 of KChIP2 are also required for sarcolemmal localization in adult atrial and ventricular myocytes (Murthy et al., 2019), although direct effects of KChIP2 S-acylation on K^+^ current in cardiomyocytes have not been evaluated.

## Phospholamban

Phospholamban is a small 52 amino acid SR membrane protein that interacts with and represses the activity SERCA2a, thereby reducing transport of cytosolic Ca^2+^ back into the SR. Upon phosphorylation by cAMP-dependent protein kinase (PKA) downstream of β-adrenergic stimulation, phospholamban relieves its inhibition of SERCA2a resulting in enhanced Ca^2+^ reuptake into the SR, restoration of diastolic cytosolic Ca^2+^ levels, and myofilament relaxation (MacLennan and Kranias, 2003; Figure 2). Thus, phospholamban functions as an instrumental regulator of cardiac contraction and relaxation. In COS7 cells, phospholamban is S-acylated at Cys36 (Figure 2) by zDHHC16, a predominantly ER-localized enzyme (Li et al., 2002; Abrami et al., 2017), which enhances its phosphorylation by PKA and oligomerization (Zhou et al., 2015). Deletion of zDHHC16 in mice results in developmental defects of the eye and heart, including ventricular non-compaction cardiomyopathy and abnormal cardiomyocyte nuclear morphology, and mortality at 1 day of age in *Zdhhc16*^–/–^ pups that are born alive (Zhou et al., 2015). Importantly, *Zdhhc16*^–/–^ hearts have reduced phospholamban interaction with PKA and phospholamban phosphorylation (Zhou et al., 2015). Moreover, deletion of phospholamban partially rescues cardiomyocyte nuclear dysmorphology in postnatal *Zdhhc16*^–/–^ hearts (Zhou et al., 2015), suggesting zDHHC16 functionally regulates phospholamban *in vivo*. Further studies are needed to substantiate regulation of phospholamban by zDHHC16 in the adult heart and test effects on Ca^2+^ handling and cardiac contractility, which would be greatly aided by generation of mice with cardiomyocyte-specific zDHHC16 deletion or a knock-in mutation of the PLN S-acylation site (Cys36) to further interrogate phospholamban modification *in vivo*.

## Junctophilin-2

Junctophilin-2 is a structural protein that tethers portions of the T-tubule sarcolemma to the SR membrane thereby forming junctional membrane complexes that enable efficient coupling of Ca^2+^ influx from the T-tubular L-type voltage-dependent Ca^2+^ channel (Ca_v_1.2) to activate RyR2 and evoke SR Ca^2+^ release and subsequent myofilament contraction (Takeshima et al., 2015; Figure 2). Junctophilin-2 is required to maintain T-tubule structure, RyR2 function, and is a requisite component of the excitation-contraction coupling machinery (Van Oort et al., 2011; Chen et al., 2013; Guo et al., 2014; Zhang et al., 2014). Not surprisingly, mutations in junctophilin-2 are associated with cardiomyopathy, arrhythmia, and heart failure (Beavers et al., 2013; Takeshima et al., 2015; Vanninen et al., 2018; Jones et al., 2019). Junctophilin-2 is a large, nearly 700 amino acid, predominantly cytoplasmic protein containing N-terminal MORN domains that associate with the plasma membrane and a C-terminal transmembrane domain embedded in the SR (Takeshima et al., 2015; Figure 2). However, mechanisms underlying junctopilin-2-dependent regulation of dyad architecture are not known. A recent study suggests junctophilin-2 is S-acylated in cardiomyocytes (Jiang et al., 2019; Figure 2). In COS7 cells, this allows for association with lipid-rafts in the plasma membrane and stabilizes ER-plasma membrane junctions (Jiang et al., 2019). Future studies are needed to determine which cysteines of junctophilin-2 are modified in cardiomyocytes and how this alters its association with the SR and sarcolemma, Ca^2+^ cycling, and T-tubule morphology.

## Other Ion Channels and Regulators

Recent studies have uncovered S-acylation of a number of ion channels and regulatory proteins in cardiomyocytes with divergent effects on protein function and electrophysiology, suggesting additional unidentified S-acylation events contribute to regulation of cardiac electrophysiology. Although Na_v_1.5 activity is regulated by S-acylation (Pei et al., 2016), it is not known whether any of its auxiliary β subunits are S-acylated. Na_v_-β1 (encoded by *SCN1B*) contains a putative S-acylation site at its intracellular juxtamembrane Cys162 (Brackenbury and Isom, 2011; Edokobi and Isom, 2018) that warrants further investigation into potential effects on Na_v_1.5 activity. In contrast, there is intricate knowledge of how S-acylation of the auxiliary protein phospholemman modulates Na^+^ pump activity (Howie et al., 2014), yet it is not clear if the α and β subunits of Na^+^/K^+^-ATPase themselves are S-acylated in cardiomyocytes and if this affects pump activity. Despite recent evidence that KChIP2 is S-acylated in cardiomyocytes (Murthy et al., 2019) and the importance of K_v_4 channels in cardiomyocyte electrophysiology, it is not known if K_v_4 or other K^+^ channels, such as the rapidly activating delayed rectifier K^+^ channel (I_Kr_), undergo S-acylation and whether this impacts trafficking, recycling, gating, or inactivation. Coupling of large conductance calcium- and voltage- activated potassium channels (BK channels) is regulated by S-acylation (Duncan et al., 2019), yet the role of this in the mechanosensing functions of BK channels in ventricular myocytes (Zhao et al., 2017) is not clear. Moreover, except for phospholamban (Zhou et al., 2015), modification of SR Ca^2+^ handling proteins by S-acylation in cardiac myocytes has not been reported, although SERCA1a, RyR1, and Ca_v_1.1 undergo S-acylation in skeletal muscle (Chaube et al., 2014), suggesting SERCA2a, RyR2, and/or Ca_v_1.2 could be similarly regulated in the heart. Additionally, in HEK cells, S-acylation of Cys3 and Cys4 of the β_2__*a*_ subunit of the L-type calcium channel is required for its plasma membrane targeting and modulation of Ca_v_1.2 current (Chien et al., 1996, 1998), suggesting this mechanism could regulate L-type calcium channel current and excitation-contraction coupling in cardiomyocytes.

Several chaperones and cytoskeletal proteins critical for trafficking and localization of ion channels to the cell surface are modified and functionally regulated by S-acylation and it is unclear if this contributes to cardiac physiology or pathogenesis of arrhythmogenic channelopathy disorders. For example, ankyrin-G is a large cytoskeletal protein whose membrane localization and function are dictated by S-acylation in epithelial cells (He et al., 2012, 2014). Ankyrin-G is required for proper targeting of Na_v_1.5 to the cardiomyocyte intercalated disc and loss of ankyrin-G results in lethal dilated cardiomyopathy, bradycardia, and hypersensitivity to cardiac arrhythmia (Makara et al., 2014, 2018). Moreover, the highly homologous scaffolding protein ankyrin-B, which contains a homologous Cys to the ankyrin-G acylation site, is necessary for targeting Na^+^/K^+^-ATPase and NCX to the cardiomyocyte T-tubule. Mutations in ankyrin-B result in “ankyrin-B syndrome” characterized by arrhythmogenic cardiomyopathy and sudden death (Mohler et al., 2003, 2004; Roberts et al., 2019). However, it is unclear if S-acylation regulates ankyrins or other ion channel targeting proteins in the heart.

Recent work has characterized a cargo-dependent, adaptor-independent endocytosis pathway termed massive endocytosis (MEND) in which large portions of the plasma membrane are internalized in response to reoxygenation of anoxic cardiac muscle (Lin et al., 2013). This unique form of endocytosis requires the activity of the plasma membrane enzyme zDHHC5 downstream of metabolic stress and mitochondrial-mediated activation of programed necrosis (Lin et al., 2013). Interestingly, S-acylation of both phospholemman and NCX1 promote MEND in response to pharmacological activation of G-protein signaling or mitochondrial Ca^2+^ overload, suggesting the presence of specific S-acylated cargo in sarcolemmal microdomains may induce this unique endocytic pathway (Lin et al., 2013; Reilly et al., 2015; Fuller et al., 2016). Further examination is required to determine the extent to which MEND occurs in the heart and participates in the response to ischemia-reperfusion injury and the activity and/or internalization cardiomyocyte ion channels, transporters, and regulatory proteins *in vivo*.

## Signaling Proteins

S-acylation is emerging as a potent regulator of intracellular signal transduction with implications for many pathophysiologies. For example, the epidermal growth factor receptor (EGFR) is regulated by S-acylation (Runkle et al., 2016; Figure 1), which directly modulate signal transduction in cancer cells. Signaling by G-protein coupled receptors (GPCRs) mediates many critical cardiac physiological responses, including contractility and hypertrophic signaling (Bristow et al., 1990; Feldman and Bristow, 1990; D’Angelo et al., 1997; Paradis et al., 2000). S-acylation of many GPCRs functionally regulates G-protein signaling *in vivo* (Liu et al., 2012; Adachi et al., 2016; Chen et al., 2017; Chen S. et al., 2019; Figure 1), yet the effects of GPCR S-acylation on receptor trafficking and signaling in cardiac physiology and disease have not been fully elucidated. Moreover, the majority of G-protein α subunits that transduce signals from activated GPCRs are S-acylated on their N-terminus (Linder et al., 1993; Oldham and Hamm, 2008) and a recent study suggests the plasma membrane-localized enzyme zDHHC5 regulates Gα_s_ and Gα_i_ S-acylation in cardiomyocytes in response to β-adrenergic stimulation (Chen J.J. et al., 2019). However, previous reports indicate that Gα protein S-acylation is catalyzed by Golgi-localized zDHHC enzymes (Tsutsumi et al., 2009). Thus, the kinetics and putative regulation of S-acylation of the many G-protein α subunits by the several zDHHC enzymes expressed in cardiomyocytes and resultant effects on transducing GPCR signals, cardiac contractility, and hypertrophy require further investigation. Furthermore, the role of acyl protein thioesterases in regulating dynamic S-acylation of signaling proteins in cardiomyocytes is virtually unexplored. Many other important cardiac signaling proteins, including regulator of G-protein signaling (RGS) proteins (Zhang and Mende, 2011), small GTPases (Navarro-Lerida et al., 2012; Modica et al., 2017; Figure 1), and kinases (Shenoy-Scaria et al., 1994; Runkle et al., 2016) are also regulated by S-acylation, warranting further investigation into the potential functions of these modifications in cardiac signal transduction and physiology. Thus, future studies into the modulation of receptors, kinases, GTPases, and other signaling molecules by S-acylation in the heart will aid in the discovery of novel signal transduction mechanisms that contribute to cardiac homeostasis and pathogenesis.

## Discussion

S-acylation represents a new frontier in post-translational modifications that can acutely and dynamically control membrane protein function and signal transduction. Its wide-ranging physiological effects clearly extend to cardiomyocytes and myocardial physiology. It is becoming increasingly evident that S-acylation plays instrumental roles in cardiac physiology although more *in vivo* data on cardiac function and disease in the context of genetic or pharmacological manipulation of zDHHC enzymes, acyl protein thioesterases, or specific S-acylation modifications will greatly enhance our understanding of S-acylation-mediated regulation in the heart. Recent advances in techniques to detect S-acylation (Forrester et al., 2011; Zaballa and Van Der Goot, 2018), including in tissue samples, will propel this field going forward. Protein cysteines can also undergo oxidation and other post-translational modifications such as glutathionylation and S-nitrosylation that can reciprocally and competitively regulate substrate function (Ho et al., 2011; Burgoyne et al., 2012; Shipston, 2014), which also necessitates careful interpretation of studies using cysteine mutagenesis. Indeed, much research remains to be conducted to understand how specific S-acyl modifications modulate protein function in cardiomyocytes and the broader impact on cardiac physiology and disease.

Pharmacological tools to manipulate zDHHC enzymes and substrate S-acylation will prove incredibly useful for basic science and potentially as novel therapeutics. The development of small molecule inhibitors with specificity for individual zDHHCs and *in vivo* efficacy continues to be a challenge but would facilitate the interrogation of the role of these enzymes in specific cell types, signaling pathways, cellular processes, and diseases and ultimately have potential for translation to the clinic. Recently, nitro-containing compounds (nitrofurans and nitro-fatty acids) have been discovered that irreversibly alkylate S-acylation sites on stimulator of interferon genes (STING) thereby inhibiting S-acylation and activation of STING (Haag et al., 2018; Hansen et al., 2018). These compounds are being pursued clinically for the treatment of autoinflammatory diseases (Haag et al., 2018; Hansen et al., 2019) and represent a new paradigm for targeted inhibition of substrate S-acylation with small molecule inhibitors.

Genetic tools and proteomics will greatly advance our understanding of the roles of S-acylation in cardiac biology and disease. Generation of *in vivo* mouse models including LoxP-targeted alleles to delete specific zDHHC enzymes in cardiac myocytes and non-myocyte cell populations (i.e., fibroblasts, immune cells) of the heart, cardiac-specific zDHHC gain-of-function transgenic mice, and knock-in mutations for specific substrate S-acylation sites, will prove invaluable in elucidating functional effects of S-acylation-dependent regulation on cardiac electrophysiology, protein trafficking, and signal transduction in cardiac physiology and disease. Moreover, proteomic studies to interrogate alterations in the cardiac palmitoylome in models with manipulated zDHHC enzyme activity and models of cardiac disease will identify novel S-acylated proteins in the heart, their cognate zDHHC enzymes, and modifications that are altered in the hypertrophied and failing heart, which will facilitate the discovery of novel molecular mechanisms participating in cardiac disease. Thus, future work will combine biochemical and molecular analyses of S-acylation modifications in cardiac cells with *in vivo* assessment of cardiac physiology and disease, which will ultimately enable a comprehensive understanding of mechanisms and functions of S-acylation in cardiac pathophysiology.

## Author Contributions

All authors contributed to the conception, writing, and editing of the manuscript.

## Conflict of Interest

The authors declare that the research was conducted in the absence of any commercial or financial relationships that could be construed as a potential conflict of interest.
